# Expression of angiogenic basic fibroblast growth factor, platelet derived growth factor, thrombospondin-1 and their receptors at the porcine maternal-fetal interface

**DOI:** 10.1186/1477-7827-9-5

**Published:** 2011-01-17

**Authors:** Andrew K Edwards, Marianne J van den Heuvel, Jocelyn M Wessels, Jonathan LaMarre, B Anne Croy, Chandrakant Tayade

**Affiliations:** 1 Department of Biomedical Science, University of Guelph, Guelph, ON N1G 2W1, Canada; 2 Department of Microbiology and Immunology, University of Western Ontario, London, ON N6A 5C1, Canada; 3 Department of Anatomy and Cell Biology, Queen's University, Kingston, ON K7L 3N6, Canada

## Abstract

**Background:**

Commercial swine breeds in North America undergo two waves of spontaneous fetal loss; one during peri-attachment and another during mid-gestation. Although an exact mechanism for this loss is not known, deficits in vasculature at the attachment sites appear to be a major cause. We hypothesized that a balance between pro-angiogenic and anti-angiogenic factors is needed at the maternal-fetal interface for successful conceptus development. Six selected members of the pro-angiogenic fibroblast growth factor (FGF) and platelet derived growth factor (PDGF) families and anti-angiogenic factor thrombospondin-1 (TSP-1) and its receptor CD36 were quantified and localized at the porcine maternal-fetal interface at early and midgestation time points.

**Methods:**

Mesometrial endometrium was collected from non-pregnant gilts (n = 8). Endometrial and chorioallantoic membrane samples were collected from healthy and arresting conceptus attachment sites at gestation day (gd) 20 (n = 8) and gd 50 (n = 8). At gd20 arresting conceptus attachment sites were distinguished by decreased vasculature of the placental membranes and decreased conceptus size. At gd50 arresting conceptuses attachment sites were identified by smaller conceptus length and weight measurements. Quantitative real time PCR was used to determine relative transcript levels of genes of interest, and cellular localization was determined by immunohistochemistry in paraffin embedded endometrial sections.

**Results:**

At gd20, endometrial samples from arresting conceptuses had elevated transcripts for bFGF, and PDGF-bb than healthy sites (p < 0.05). At gd50, bFGF, FGFR2, and CD36 were more abundant at arresting than at healthy conceptus attachment sites (p < 0.05). Chorioallantoic membrane from arresting conceptus attachment sites at gd20 had elevated transcripts for bFGF, FGFR1, FGFR2 and CD36 compared with healthy sites (p < 0.05). FGFR2 transcripts were more abundant in chorioallantoic membrane from arresting conceptuses at gd 50 (p < 0.05). Immunohistochemical localization of selected pro- and anti-angiogenic factors and receptors revealed their abundance in the luminal epithelium, uterine glands and perivascular areas of endometrium at gd20 and gd50.

**Conclusions:**

We provide comprehensive analysis of pro and anti-angiogenic factors at the porcine maternal fetal interface during early and mid-pregnancy. At mRNA levels, the majority of pro-angiogenic factors investigated were elevated at the sites of fetal arrest. These observations contrast with our previous findings of decreased Vascular Endothelial Growth Factor (VEGF) family members at arresting sites, and suggest that the bFGF family functions as a compensatory survival mechanism when major angiogenic proteins are decreasing at the sites of fetal arrest.

## Background

Prenatal mortality is a prime concern for commercial pork industry in North America. Thirty to forty percent of conceptuses are lost during gestation [1]. This spontaneous fetal loss is found in two waves, a peri-attachment wave of conceptus loss (gd 10-30) and a mid gestational wave of fetal loss (gd 50-70) [1-4]. Since only 1.3% of conceptuses have a gross chromosomal abnormality, conceptus genetics is unlikely to account for more than a minor proportion of the fetal losses [5]. Although exact mechanisms for the fetal loss are still unknown, angiogenesis appears to be crucial in successful development of conceptuses through gestation.

Angiogenesis is defined as the formation of blood vessels from pre-existing blood vessels. Swine, a species with an epitheliochorial form of placentation, undergo extensive angiogenesis at the maternal-fetal interface to meet the nutrient requirements of the developing conceptus [6]. Investigations of the porcine maternal-fetal interface at time points representative of the peri-attachment [3,4,7] and mid-gestational [7] stages of conceptus loss showed that conceptuses undergoing growth arrest had decreased endometrial vasculature compared to their healthy counterparts. We postulate that a deficit in vascular development at the maternal-fetal interface may play a participatory role in the conceptus loss that occurs during porcine pregnancy. Previously, we have reported decreased transcripts of the prime pro-angiogenic molecule VEGF and two of its receptors, VEGFRI and VEGFRII, in endometrial lymphocytes, endometrium and trophoblast associated with arresting conceptus attachment sites [3,4,7]. Although there was no direct evidence that the arresting conceptuses identified at early or mid-gestation will be lost later during gestation, our studies provided first evidence [3,4,7] that dysregulation in angiogenesis at the maternal-fetal interface is a prime cause leading to growth arrest of developing conceptuses. Given that angiogenesis is a complex process which is regulated through a number of alternate pathways, we extended our investigations of the maternal-fetal interface in relation to pregnancy success or failure.

Basic Fibroblast Growth Factor (bFGF) is a pro-angiogenic molecule, a potent mitogen of endothelial cells and provides the initial stimulus needed for angiogenesis [8]. It is highly expressed in endometrium during rat, human and primate pregnancy [9-12]. In porcine endometrium, bFGF and its two angiogenic receptors (FGFR1 and FGFR2) are expressed at the beginning of pregnancy (gd10) when extensive angiogenesis is occurring [13]. Basic fibroblast growth factor and FGFR1 are localized in the luminal epithelium, stroma and glands in porcine endometrium [13-15]. FGF-9 is highly upregulated in pregnant porcine endometrium, and its localization to the glandular epithelium indicates it could act as an important embryonic growth factor [16].

Platelet derived growth factor-bb, another pro-angiogenic growth factor, can stimulate endothelial cells to form nascent vascular networks and recruit mural cells to stabilize developing blood vessels [17,18]. PDGF-bb's two main angiogenic receptors, PDGFRα and PDGFRβ, are involved in different aspects of angiogenesis; PDGFRα in the stimulation of endothelial cells, and PDGFRβ in the recruitment of mural cells [19,20]. During pregnancy, PDGFs are actively expressed on both sides of the maternal-fetal interface in humans [21,22]. Platelet Derived Growth Factor-bb and PDGFRβ play an essential role in normal murine placental development. Knockouts for either show abnormal labyrinthine formation, reduced labyrinthine trophoblast, and inherently dilated blood vessels that lack pericytes [19,20,23]. Distribution of PDGFRα and β has been well characterized [24] at the porcine maternal-fetal interface at the beginning of pregnancy (gd7-12).

Thrombospondin-1 (TSP-1) is a potent anti-angiogenic factor and effects angiogenesis by directly binding CD36 expressed on endothelial cells, as well as through growth factors, cytokines and proteases [25]. Thrombospondin-1 has the ability to inhibit endothelial cell DNA synthesis, leaving the cells unable to proliferate [26]. Thrombospondin-1 can directly bind VEGF, leading to internalization through the low density lipoprotein receptor-1 receptor [27]. Thrombospondin-1 also regulates the activity and bioavailability of matrix metalloproteinases, which in turn, regulate VEGF bioavailability by releasing matrix bound VEGF [28]. In humans, TSP-1 is expressed by endometrial stromal cells [29]. CD36, the primary TSP-1 anti-angiogenic receptor, is also expressed in human endometrium [30]. Thrombospondin-1 and CD36 have never been investigated at the maternal-fetal interface of a species with epitheliochorial placentation.

The purpose of this study was to determine whether changes in pro- and anti-angiogenic factors are associated with spontaneous fetal loss in North American swine. We hypothesized that a balance between pro-angiogenic (FGF and PDGF family members) and anti-angiogenic factors (TSP-1 and CD36) is required at the porcine maternal-fetal interface to promote successful conceptus development.

## Methods

### Porcine tissue sample collection

Porcine endometrial and chorioallantoic membrane samples were obtained from specific pathogen-free Yorkshire gilts housed at the Arkell Swine Research Station of the University of Guelph. First time cycling gilts were slaughtered and uteri collected from non-pregnant (NP) (n = 8, diestrus), gd20 (n = 8) and gd50 (n = 8) stages. Immediately following slaughter, uteri were transported on ice, to an RNAse decontaminated laboratory. Random samples of mesometrial endometrium were collected from non-pregnant uteri. At gd20 and gd50, healthy and arresting littermates were identified and endometrial and chorioallantoic membrane samples were taken from the attachment sites [3,4,7]. Arresting conceptuses were identified by decreased size and lower placental vascularity at gd20, and decreased conceptus length and weight measurements at gd50 [3]. Samples were collected from three healthy attachment sites, and all arresting conceptus attachment sites per uterus. Each sample was divided into three portions; one was snap-frozen at -80°C for RNA and protein extraction, another was frozen at -80°C in OCT cryomatrix for cryosectioning, and the last was placed in 4% paraformaldehyde for paraffin embedded immunohistochemistry.

### RNA extraction from endometrial and chorioallantoic membrane samples

RNA extraction was performed using RNAeasy Mini Kit (Qiagen Sciences, Mississauga, ON, Canada) as per manufacturer's instructions. Thirty mg of tissue was disrupted and homogenized with Buffer RLT provided with the kit containing β-mercaptoethanol in a microcentrifuge tube. A rotor-stator homogenizer was used to disrupt the tissue (Fisher Scientific, Ottawa, ON). The lysate was centrifuged and 600 μL of 70% ethanol was added to the supernatant. Samples were loaded onto RNAeasy spin columns and centrifuged for 15 seconds. After two washings of spin columns with wash buffer provided with the kit, purified RNA was eluted by adding 30 μL of RNAase/DNAase free water. The Gene Quant Pro RNA/DNA calculator (Biochrome IDD, Cambridge, MA) was then used to measure RNA concentration. RNA was immediately put through first strand cDNA synthesis, or frozen at -80°C for subsequent use.

### cDNA synthesis

cDNA synthesis was performed using First-Strand cDNA Synthesis Kit as per manufacturer's instructions (GE Healthcare, Buckinghamshire, UK). RNA (1.5 μg in 20 μL) heated at 65°C for 10 minutes in GeneAMP PCR System 2700 (Applied Biosystems, Foster, CA). To this, 11 μL of the bulk first strand cDNA reaction mix, 1 μL of DTT solution and 1 μL of poly(dT) primer was added. The sample was incubated for 60 minutes at 37°C in the PCR machine. The cDNA was stored in -20°C for future use as a template for real time polymerase chain reaction.

### Quantitative real-time PCR

Primers were designed based on coding sequence homology from different mammalian species (human, mouse, rat, cow, horse and dog) usingPrimer3 http://frodo.wi.mit.edu/primer3/ program. Target genes were amplified from pooled porcine reproductive tissue cDNA with Quantitect SYBR Green I PCR mix (Qiagen) on real-time capillary based PCR system (LightCycler, Roche Diagnostics, Laval, QC). Amplification product was sequenced to confirm that primers were amplifying target genes. Following confirmation that primer sets were amplifying desired genes, relative quantification with *β-actin *as a housekeeping gene was performed using the LightCycler480 (Roche Diagnostics, Laval, QC). Data were analyzed using LightCycler480 RelQuant software.

### Immunohistochemistry in paraffin embedded porcine endometrial sections

Paraffin-embedded endometrial sections were cut at 7 μm and placed on positively charged microscope slides (Superfrost Plus, Fisher Scientific, Ottawa, ON). Sections were deparaffinised by serial rinsing in 100% xylene, 100%, 90% and 70% ethanol, and water. Antigen retrieval was performed by boiling the section in citrate buffer (pH 6.5) for five minutes. The sections were covered with Peroxidase Block (DAKO, Carpenteria, CA) for five minutes and rinsed with Tris-buffered saline (TBS). Non-specific blocking was performed with serum-free protein block (DAKO, Carpenteria, CA). Primary antibody for the proteins of interest was applied to sections with concentrations varying from 1 μg/ml - 4 μg/ml (as titrated in the laboratory, antibody concentrations and source are provided in Table 1) for 1 hour. Negative slides were treated identically except isotype control antibody was used instead of primary antibody. Sections were rinsed and placed in a bath of TBS containing 0.05% Tween 20 (TBST) and then incubated with a biotinylated secondary antibody 0.2 μg/ml (DAKO, Carpenteria, CA) for 15 minutes. After rinsing in TBST, sections were covered with streptavidin conjugated to horseradish peroxidise (DAKO, Carpenteria, CA) for 15 minutes. Final color was developed using DAB+ chromogen and substrate (DAKO, Carpenteria, CA) for 5 minutes. Slides were counterstained with haematoxylin for three minutes and dehydrated in serial washes of increasing ethanol concentrations (70-100%) followed by xylene. Slides were examined using a Leica DMLB microscope (Leica Microsystems Inc., Richmond Hill, ON). Images were captured using QCapture Pro software (QImaging Corporation, Surrey, BC).

**Table 1:** Antibody sources and concentrations used for immunohistochemistry experiments

Antibody (Abcam)	Concentration
Rabbit Polyclonal to FGFR1(ab10646)	1 μg/ml
Rabbit Polyclonal to FGFR2 (ab10648)	4 μg/ml
Rabbit Polyclonal to PDGF-bb (ab23914)	4 μg/ml
Mouse Monoclonal to PDGFRβ (ab10847)	4 μg/ml
Mouse Monoclonal to Thrombospondin-1 (ab1823)	4 μg/ml
Rabbit Polyclonal to CD36 (ab36977)	4 μg/ml

### Statistical analysis

Statistical analysis of quantitative RT-PCR was done using Sigma Stat 3.5 software. Data was logarithmically transformed before an ANOVA on Ranks test was performed. If the data were normally distributed, and had equal variance, a one way ANOVA was performed instead. For ANOVA on Ranks, a Dunn's post test was used. For a one way ANOVA, a Holm-Sidak post test was used. A p-value < 0.05 was considered significant.

## Results

### Expression of bFGF, FGFR1, FGFR2 mRNAs level and immunolocalization

**Figure 1:**
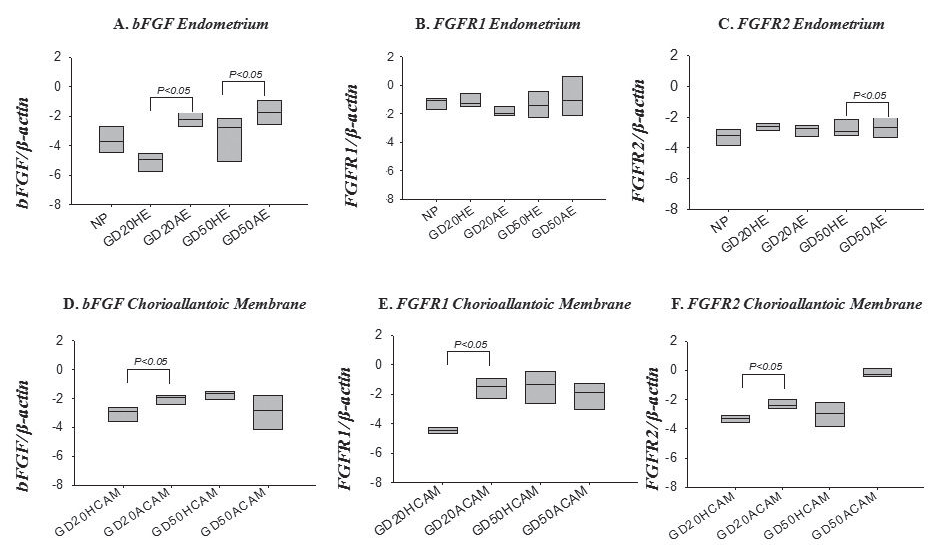
**mRNA expression of bFGF and its receptors at the porcine maternal-fetal interface**. bFGF (A, D), FGFR1 (B, E), and FGFR2 (C, F) mRNA transcripts relative to β-actin transcripts were quantified in non-pregnant (NP) endometrium, and gd20 and gd50 endometrium (A, B, C) and chorioallantoic membrane (D, E, F) from healthy (HE, HCAM) and arresting (AE, ACAM) conceptus attachment sites. Log transformed data was analyzed by ANOVA, p < 0.05 was considered significant. ACAM: arresting conceptus chorioallantoic membrane; HCAM: healthy conceptus chorioallantoic membrane; HE; healthy endometrium; AE: arresting endometrium.

Basic fibroblast growth factor, FGFR1, and FGFR2 were transcribed in non-pregnant endometrium as well as endometrium and chorioallantoic membrane at gd20 and 50 (Figure 1). Transcripts of bFGF were higher in endometrium from arresting conceptus attachment sites compared to tissue from healthy sites at both gd20, and gd50 (p < 0.05) (Figure 1A). No significant differences were found for FGFR1 between endometrial tissue from healthy and arresting conceptus attachment sites at either time points (Figure 1B). For FGFR2, endometrial tissues from arresting conceptus attachment sites had elevated FGFR2 transcript levels compared to healthy at gd50 (Figure 1C) (p < 0.05); no difference was seen at gd20.

Chorioallantoic membrane from arresting conceptus attachment sites demonstrated elevated transcript levels of bFGF in comparison with healthy at gd20 (p < 0.05; Figure 1D). Similarly Chorioallantoic membrane from arresting gd20 conceptus attachment sites had elevated transcript levels of FGFR1 and FGFR2 compared to tissue from healthy sites (Figure 1D, E p < 0.05). At gd50, no significant difference was observed between healthy and arresting conceptus attachment sites for these three genes (Figure 1D, E, F).

**Figure 2:**
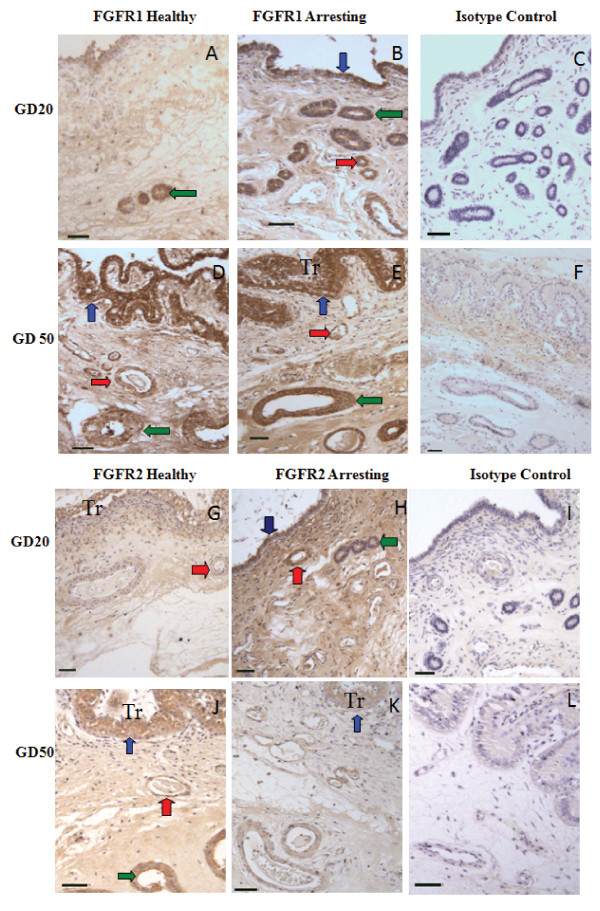
**Immunohistochemical localization of FGFR1 and FGFR2**. FGFR1 (A-F) and FGFR2 (G-L) expression in endometrium from healthy (A, D, G, J) and arresting (B, E, H, K) conceptus attachment sites at gd20 and gd50. Expression of both receptors was associated with blood vessels (red arrows), luminal epithelium (blue arrows), and uterine glands (green arrows). Trophoblast (Tr) are visible in some sections. Size bars represent 100 μm.

Fibroblast growth factor receptors 1 and 2 expression was confirmed in endometrium from healthy and arresting conceptus attachment sites at gd20 and gd50 by immunohistochemistry (Figure 2). The receptors were localized in the endometrial luminal epithelium, blood vessels, and uterine glands (Figure 2).

### Expression of PDGF-bb, PDGFRα, and PDGFRβ transcript levels and immunohistochemistry

**Figure 3:**
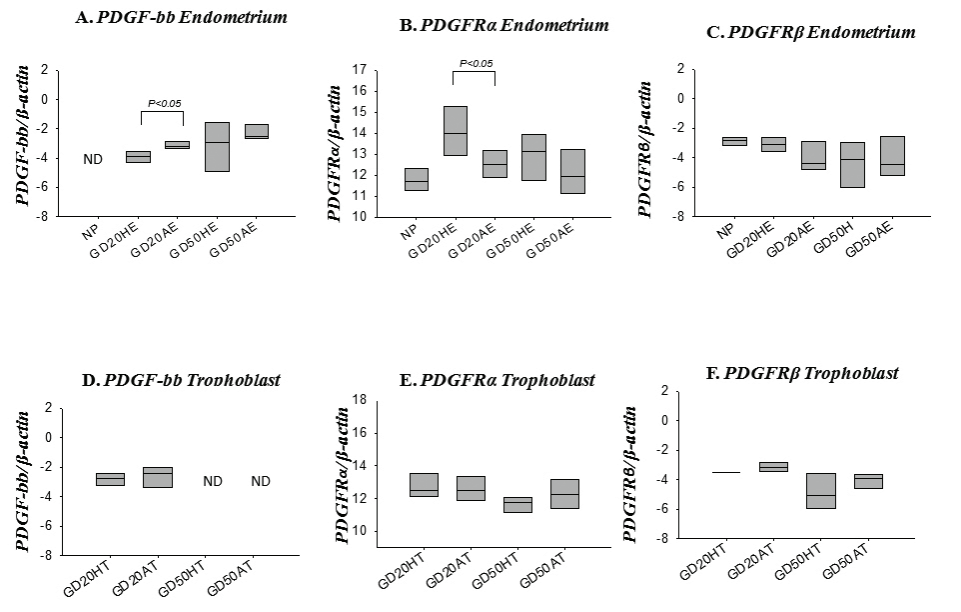
**mRNA expression of PDGF-bb and its receptors at the porcine maternal-fetal interface**. PDGF-bb (A, D), PDGFRα (B, E), and PDGFRβ (C, F) mRNA transcripts were quantified in non-pregnant endometrium and gd20 and gd50 endometrium and chorioallantoic membrane from healthy (HE, HCAM) and arresting (AE, ACAM) conceptus attachment sites. Transcript levels are relative to β-actin. ND= Not detected. Data was log transformed before analysis by ANOVA, p < 0.05 were considered significant. ACAM: arresting conceptus chorioallantoic membrane; HCAM: healthy conceptus chorioallantoic membrane; HE; healthy endometrium; AE: arresting endometrium.

Transcript levels of PDGF-bb were higher in endometrial tissue from arresting conceptus attachment sites compared to endometrial tissue from healthy sites at gd20 (p < 0.05), but were unchanged at gd50 (Figure 3A). Transcripts for both receptors (PFGFRα and β) were present in non-pregnant endometrium, endometrium associated with attachment sites, and gd20 and gd50 chorioallantoic membrane (Figure 3B, C, E, F). No PDGF-bb transcripts were detected in non-pregnant endometrium or in gd50 chorioallantoic membrane. An opposite trend was seen for PDGFRα expression, such that tissue from healthy conceptus attachment sites had elevated PDGFRα transcript levels compared to arresting sites at gd20 (p < 0.05; Figure 3B). Platelet derived growth factor receptor-β transcript levels did not differ between endometrium at healthy and arresting conceptus attachment sites (Figure 3C). Platelet Derived Growth Factor family member transcript levels did not differ between tissue from healthy and arresting conceptus attachment sites for gd20 or gd50 chorioallantoic membrane (Figure 3D, E, F).

**Figure 4:**
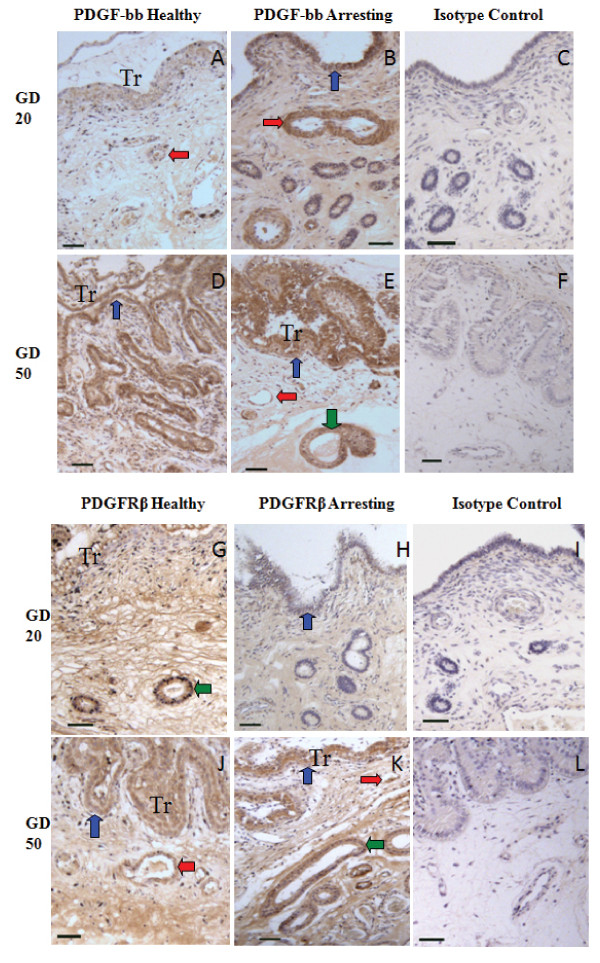
**Immunohistochemical localization of PDGF-bb and PDGFRβ**. PDGF-bb (A-F) and PDGFRβ (G-L) expression in endometrium from healthy (A, D, G, J), and arresting (B, E, H, K) attachment sites at gd20 and 50. Expression of both ligand and receptor was associated with blood vessels (red arrows), luminal epithelium (blue arrows), and uterine glands (green arrows). Trophoblasts (Tr) are visible in some sections. Size bars represent 100 μm.

Platelet derived growth factor-bb and PDGFRβ expression was confirmed in endometrial tissue associated with healthy and arresting conceptus attachment sites by immunohistochemistry (Figure 4). Expression of PDGF-bb was localized to the luminal epithelium, blood vessels, and uterine glands (Figure 4A, B, D, E). Immunostaining of PDGF-bb appeared more intense in tissue from arresting conceptus sites at gd20 especially in the luminal epithelium and in blood vessels (Figure 4A, and 4B). Platelet Derived Growth Factor Receptor-β was localized to the luminal epithelium, uterine glands, and blood vessels (Figure 4G, H, J, K).

### Anti-angiogenic factor TSP-1 and its receptor CD36 are present at the porcine maternal-fetal interface

**Figure 5:**
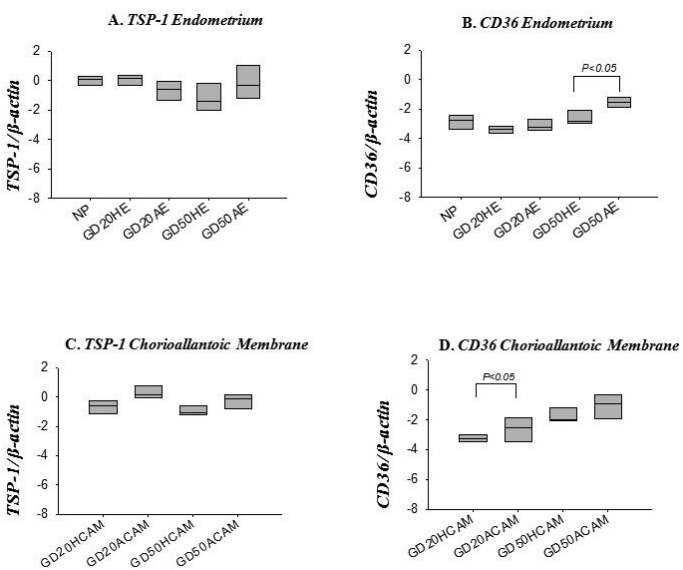
**mRNA expression of TSP-1 and CD36 at the porcine maternal-fetal interface**. TSP-1 (A), and CD36 (B) mRNA transcripts in non-pregnant endometrium and gd20 and gd50 endometrium (A,C) and chorioallantoic membrane (B, D) were quantified from healthy (HE, HCAM) and arresting (AE, ACAM) conceptus attachment sites. Transcript levels are relative to β-actin. Data was log transformed and analyzed by ANOVA. A p < 0.05 was considered significant. ACAM: arresting conceptus chorioallantoic membrane; HCAM: healthy conceptus chorioallantoic membrane; HE; healthy endometrium; AE: arresting endometrium.

**Figure 6:**
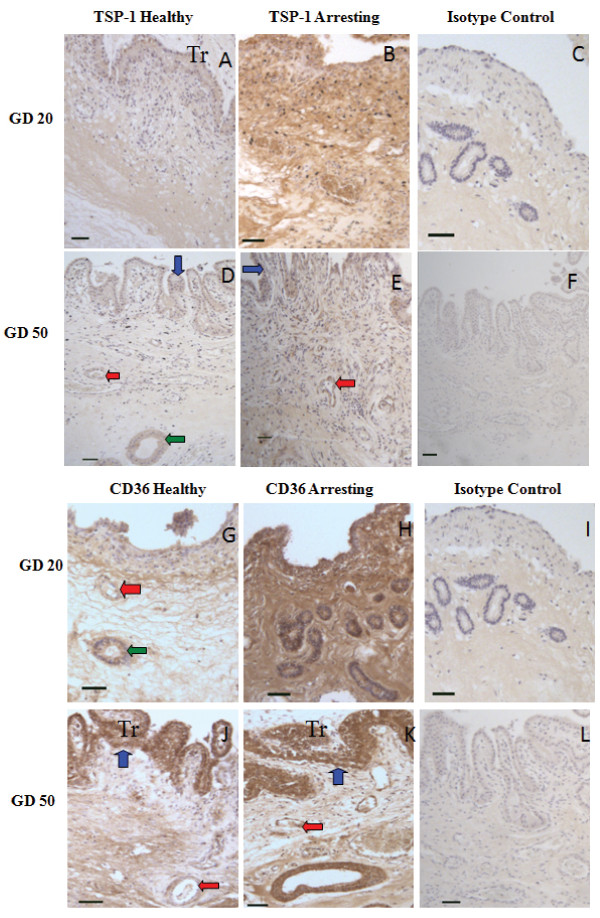
**Immunohistochemical localization of TSP-1 and CD36**. TSP-1 (A-F) and CD36 (G-L) were immunolocalized in endometrium at healthy (A, D) and arresting (B, E) conceptus attachment sites gd20 and 50. Expression of ligand and receptor was localized to blood vessels (red arrows), luminal epithelium (blue arrows), and uterine glands (green arrow). Size bars represent 100 μm.

Both TSP-1 and CD36 were transcribed in non-pregnant endometrium, as well as in endometrium and chorioallantoic membrane at gd20 and 50 (Figure 5). No difference was found for transcripts of TSP-1 between endometrial tissue from healthy and arresting conceptus attachment sites at gd20 or gd50 (Figure 5A). In endometrium at gd50, arresting conceptus attachment sites had elevated CD36 transcript levels compared to healthy sites (p < 0.05), but no difference was found at gd20 or between endometrial samples from non-pregnant and pregnant pigs (Figure 5B). Thrombospondin-1 transcript levels were not significantly different between chorioallantoic membrane from healthy and arresting conceptus attachment sites at gd20 or gd50 (Figure 5C). Chorioallantoic membrane samples from arresting conceptus attachment sites had elevated transcript levels of CD36 compared to tissue from healthy sites at gd20 (p < 0.05), but not at gd50 (Figure 5D). Both TSP-1 (Figure 6A, B, D,E) and CD36 (Figure 6G, H, J, K) expression was localized to the luminal epithelium, uterine glands and blood vessels.

## Discussion

Successful mammalian pregnancy requires a substantial increase in blood flow at the maternal-fetal interface to meet the nutrient demands of the growing conceptus. Various species have evolved various mechanisms to accomplish this increase in blood flow. In some species, like human and mice, blood flow is increased by remodelling endometrial arteries. In swine, blood flow to the maternal-fetal interface is increased almost exclusively by endometrial and placental angiogenesis. Regulation of angiogenesis and the development of maternal and placental vasculature are of central importance to optimal conceptus development. The goal of this study was to identify and characterize the dynamic changes in angiogenic growth factor expression at the maternal-fetal interface during the first half of pregnancy, when pregnancy is most likely to fail.

We investigated the FGF and PDGF pro-angiogenic families. With the exception of PDGFRα, pro-angiogenic growth factors and receptors transcript levels were elevated at sites of fetal arrest. These findings raise an intriguing question. Why, at sites of fetal arrest, where it is well documented that there is reduced vasculature at the maternal-fetal interface [3,4,7] and pro-angiogenic molecules (VEGF) are found to be decreased, would members of other pro-angiogenic families have elevated transcripts? We postulate that at sites of fetal arrest, the conceptus responds to signals (or by a dearth of signal) induced by restricted vascularization, by producing transcripts of the alternate pro-angiogenic molecules bFGF and PDGFbb,thereby potentially increasing its chances of survival. This could be a survival mechanism to compensate for decreased VEGF at these sites. The increase in FGFR1 and FGFR2 transcript levels at arresting sites in chorioallantoic membrane at gd20 could also be attributed to this compensatory survival mechanism. Sufficient receptor expression would be necessary to allow bFGF to induce angiogenesis. The elevated bFGF transcript levels at these sites could also drive the elevation in receptor transcript level. In a swine model of induced endothelial cell dysfunction, administration of bFGF was able to increase FGFR1 protein expression [31].

BFGF, PDGF-bb and their angiogenic receptors have a variety of functions at maternal-fetal interface outside of angiogenesis. They are involved in tissue remodelling and function as both propagators and effectors of the inflammatory response [8,17]. The ubiquitous expression of bFGF, which has been previously localized to the luminal epithelium, uterine glands, and uterine stromal cells and corresponding extracellular matrix in pregnant porcine endometrium [14] provides further evidence that it has functions outside of angiogenesis at the porcine maternal-fetal interface. The increase in bFGF or PDGF-bb transcript levels seen at arresting attachment sites may not solely be associated with angiogenesis, but rather one of the other functions of the molecule such as tissue remodelling or effectors of an inflammatory response. Immunohistochemistry was performed to visualize whether the localization of angiogenic factors and receptors differed between healthy and arresting endometrial attachment sites. Despite repeated attempts with varying antibody concentrations or modifications in the protocol, we were unable to optimize immunostaining for bFGF. However, FGFR1 and FGFR2 were localized to the luminal epithelium, uterine stroma, uterine glands, and blood vessels in endometrium at both healthy and arresting attachment sites, similar to previous findings [13]. Similar localization was observed for PDGF-bb and PDGFRβ. The localization of PDGF-bb and PDGFRβ in perivascular areas at sites of fetal arrest, along with their ample expression in arresting tissues, indicates that inhibited stabilization of nascent blood vessels at arresting sites does not contribute to the lack of vasculature.

Anti-angiogenesis is essential for regulating growth of nascent blood vessels. To our surprise, TSP-1 transcript levels did not differ between healthy and arresting in endometrium or chorioallantoic membrane at gd20 or gd50. In contrast, the TSP-1 receptor CD36 shows elevated transcription at sites of arrest in gd20 chorioallantoic membrane and gd50 endometrium. In the absence of an increase in ligand (TSP-1), it is difficult to assess the contribution that an increase in receptor expression would have on anti-angiogenic activity. Currently, there are no reports of constitutive anti-angiogenic activity for CD36. CD36 is highly expressed on macrophages and platelets [32], where it also functions as a scavenger receptor, and is involved in the uptake of apoptotic particles [33]. Previously, we have shown elevated transcript levels for FAS/FASL at arresting attachment sites during peri-attachment, indicating the possibility of increased apoptosis [3]. If CD36 is elevated at arresting sites, it could be related to apoptosis and not anti-angiogenic activity. But a role in anti-angiogenesis is strongly suggested, since both TSP-1 and CD36 were localized to perivascular areas by immunohistochemistry. These observations point out potential anti-angiogenic mechanisms at play at the porcine maternal-fetal interface, but there is insufficient evidence to suggest that abnormal anti-angiogenic activity contributes to fetal arrest.

This study provides insight into the changes of pro-angiogenic growth factor expression during the first half of pregnancy in a species with epitheliochorial placentation. It also demonstrates for the first time that anti-angiogenic growth factors are abundantly present particularly at arresting sites at the maternal-fetal interface during porcine pregnancy.

## Competing interests

The authors declare that they have no competing interests.

## Authors' contributions

AKE carried out real time PCR and immunohistochemistry experiments, performed statistical analysis and wrote the manuscript. MJVH helped in the real-time PCR experiments and statistical analysis. JMW helped with the immunohistochemistry experiments. BAC participated in designing this work and editing manuscript. CT conceived the experimental work, designed experiments and wrote part of the manuscript. All authors read and approved the manuscript.
